# GnRHa as a treatment for letrozole-resistant recurrent adult granulosa cell tumors: A case report

**DOI:** 10.1097/MD.0000000000028343

**Published:** 2021-12-23

**Authors:** Yuan Zhuang, Shushan Zhang, Yao Liu, Hua Yang

**Affiliations:** a Department of Gynecology, The Fifth Affiliated Hospital of Sun Yat-sen University, Zhuhai, China; b Sun Yat-sen University, Zhuhai, China.

**Keywords:** gonadotropin-releasing hormone agonists, hormone therapy, recurrent adult granulosa cell tumors

## Abstract

**Introduction:**

The optimal management of recurrent ovarian granulosa cell tumors is still unknown, and hormone therapy may be an alternative for chemotherapy-resistant cases.

**Patient concerns:**

A 46-year-old woman presented with a third recurrence after primary treatment of granulosa cell tumors. She developed tumor progression and drug-induced nephritis after 6 cycles of combined treatment with cisplatin and paclitaxel for the second recurrence and failed to benefit from chemotherapy, after the third optimal cytoreduction and tumor progression after 6 months of letrozole treatment.

**Diagnosis:**

Letrozole-resistant recurrent ovarian granulosa cell tumors

**Interventions:**

Intramuscular Diphereline 3.75 mg q28d.

**Outcomes:**

Computed tomography showed the metastatic neoplasm resolved. Progression-free survival is 20 months.

**Conclusion:**

Hormone therapy may be an alternative to treat recurrent granulosa cell tumors, and gonadotropin-releasing hormone agonists may be a rescue treatment for aromatase inhibitor-resistant cases.

## 1 Introduction

Ovarian granulosa cell tumors (GCTs) constitute less than 5% of all ovarian tumors. Unlike epithelial ovarian tumors, they occur in a younger age group and are usually detected at an early stage. They follow an indolent course and are characterized by a long natural history. Due to the chance of recurrence even years after apparent clinical cure of the primary tumor, lifelong follow-up is recommended. Approximately 25% of GCTs develop recurrence, and the median time of recurrence is usually 4 to 5 years.^[1]^ Most recurrences are intraperitoneal, and complete debulking of the disease is feasible even in cases of recurrence. Postoperative chemotherapy (platinum-based) is usually administered after surgery in cases with widespread disease or after suboptimal cytoreduction. Recurrent chemoresistant, progressive non-responding GCTs, or patients with high surgical risk are ideal candidates for targeted therapy.^[2]^ During the last decade, our understanding of the molecular pathogenesis of adult GCTs has significantly improved, whereas developments in chemotherapeutic regimens and targeted therapies have remained modest. Here, we report a case of recurrent GCTs, and after three cycles of cytoreduction, we administered letrozole as postoperative treatment for 6 months. Recurrence was confirmed by radiographic findings, and letrozole resistance was considered. We then administered GnRHa treatment and achieved a clinical cure.

## 2 Case presentation

**Figure 1:**
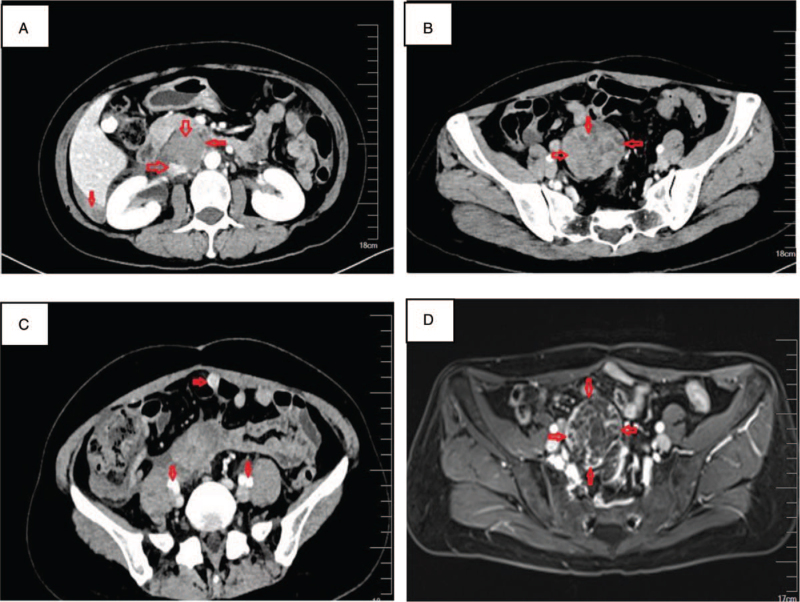
Imaging results between May 2018 and July 2019.

**Figure 2:**
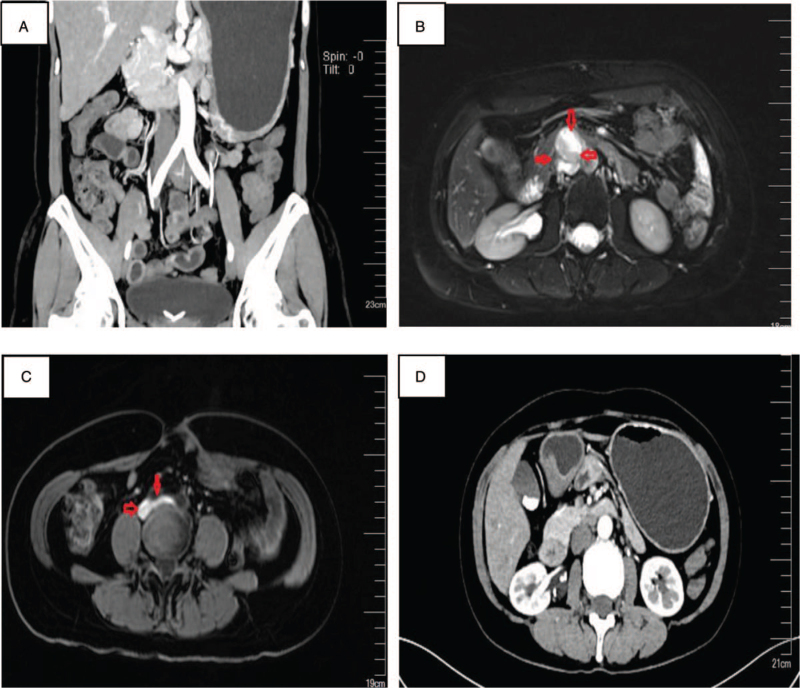
Imaging results between November 2019 and October 2021.

A 46-year-old female patient underwent a third recurrence after primary treatment for adult GCTs. Total abdominal hysterectomy and bilateral salpingo-oophorectomy with pelvic and abdominal para-aortic lymph node dissection were performed under open surgery for the Ia stage of left ovarian GCTs in 2005. During 10 years of regular postoperative follow-up, the tumor markers (including CA12-5, CA19-9, CEA, CA153, AFP, AMH, and inhibin A) and imaging evaluation were normal. In February 2017, with the complaint of lower abdominal pain with abdominal distension, nausea, vomiting, bloody stool, and other discomfort, she was referred to the Gynecology department. A mass of 73 × 62 mm was found in the pelvic midline region on a total abdominal MRI scan. Local recurrence of GCTs was considered, and laparoscopic pelvic mass resection was performed. Intraoperative exploration revealed a mass of about 8.0 × 6.0 cm in the mid-pelvic cavity with unclear boundary. Adhesion to the bowel, small intestine, and sigmoid distortion were observed. Part of the intestinal serous layer was invaded by tumor and the surgeons completely removed the mass without excising the intestines. Postoperative pathology confirmed recurrence of ovarian GCTs. Immunohistochemistry showed tumor cells: vimentin (+), CD99(+), and inhibi(+). However, the patient refused postoperative chemotherapy. In May 2018, a CT scan showed multiple masses located in the retroperitoneum, liver, and kidney recess (Fig. 1A), peritoneum, and pelvic cavity (Fig. 1B,C). Considering metastatic tumors with partial bleeding, no significant changes in pelvic and abdominal tumors were observed after treatment with paclitaxel (240 mg) and cisplatin (100 mg) for 6 cycles. Elevated serum creatinine level was consistent with drug-induced interstitial nephritis, and symptomatic treatment was administered. In July 2019, MRI scan showed multiple metastatic tumors of liver and kidney recess, pelvic wall peritoneum, and pelvic cavity with partial hemorrhage. The lesion was slightly enlarged (Fig. 1D). The third optimal cytoreduction was performed on July 16, 2019, ensuring no residual disease. Intraoperative exploration revealed 2.0 × 3.0 × 2.0 cm; Metastatic neoplasm located in the right pelvic cavity, 1.5 × 2.0 × 3.0 cm; Metastatic neoplasm in the left pelvic cavity, about 8.0 × 7.0 × 7.0 cm. Metastatic neoplasm transposited the anterior wall of the sigmoid rectum and encapsulated by the gut, infiltrative growth, multiple localized tumors located in the peritoneum. A localized tumor mass of about 5.0 × 5.0 × 4.0 cm was seen in the peritoneum of the hepatic and renal recess, and no enlarged lymph nodes were found. No significant tumor was found on the surface of liver and diaphragm. The surgeons completely removed all visible metastatic tumors with partial sigmoidectomy and intestinal anastomosis. Postoperative pathologic findings showed metastatic ovarian GCT; D99(+), CD56(+), Ki67(10%+) (Fig. 1D). Postoperative adjuvant therapy was Letrozole 2.5 mg qd. A total abdominal CT scan was reviewed in November 2019 and no abnormality was found (Fig. 2A). The patient was continued to be treated with letrozole. But in February 2020, the MRI scan showed a 3.0 × 2.5 cm metastatic neoplasm located abdominal para-aorta (Fig. 2B). Letrozole resistance was diagnosed and started treatment with Diphereline 3.75 mg IM q28d for 3 cycles. The size of the metastatic neoplasm reduced to 1.3 × 0.5 cm observed in the CT scan in August 2020 (Fig. 2C). The patient was continued to be treated with Diphereline. In October 2021, CT scan showed the resolution of metastatic neoplasm (Fig. 2D). So far, progression-free survival has reached 20 months. The current treatment program will be continued. The summary of diagnoses and interventions are as summarized in Table 1.

**Table 1:** The timeline of diagnoses and interventions.

Dates	Diagnoses	Interventions
2005	ovarian GCTs stage Ia	Total abdominal hysterectomy, bilateral salpingo-oophorectomy with pelvic and abdominal para-aortic lymph node dissection
2017-02	1st recurrence of GCTs	Laparoscopic pelvic mass resection
2018-05	2nd recurrence of GCTs	Combined paclitaxel 240 mg and cisplatin 100 mg for 6 cycles
2019-06	Progress in recurrent GCTs	Optimal cytoreduction to no residual disease,followed by Letrozole 2.5 mg qd
2020-02	Third recurrence of GCTs; Letrozole resistance	Diphereline 3.75 mg im q28d
2021-10	clinical cure	Diphereline 3.75 mg im q28d

## 3 Discussion

GCTs have a tendency for late recurrence and lead to fatality in 82% of cases. The longest reported survival time after recurrence was 40 years. Approximately 21% of patients developed recurrence, and the median time to relapse was 57.6 months (2--166 months) according to previous reports.^[3]^ The optimal management of recurrent GCTs has never been determined by randomized trials, and a combined modality of treatment, usually involving debulking of the disease followed by radiation or chemotherapy, is the norm and may prolong the DFS. Response rates for the most common combination of bleomycin, etoposide, and cisplatin varied from 37% to 83% in older studies.^[4]^ However, in the most recent series, the responses were only moderate, reaching 22% to 35%.^[5]^ It must be noted that the current evidence is based mostly on retrospective studies of non-validated GCT cohorts, presenting as a potential confounder when evaluating these responses. Combination chemotherapy with paclitaxel and carboplatin has also been used, providing the same efficacy albeit less toxicity compared with bleomycin, etoposide, and cisplatin, and the role of adjuvant chemotherapy in adult GCTs is also obscure; reasonably high response rates to platinum-based combination therapies have been reported.^[6]^ However, adjuvant chemotherapy does not seem to significantly affect patient outcomes.^[1]^ This patient developed tumor progression and drug-induced nephritis after 6 cycles of TP chemotherapy for the second recurrence and failed to benefit from chemotherapy. Therefore, after the third optimal cytoreduction, no additional adjuvant chemotherapy was administered.

The treatment of patients resistant to chemotherapy remains a difficult problem. GCTs are hormone sensitive. Compared with chemotherapy, hormone therapy has the advantages of high tolerance, long-term application, and no serious side effects. At present, this study is limited to case reports. A recent systematic review^[7]^ reviewed 31 patients from 19 studies with a total response rate of 71%, with a complete response rate of 25.8% and partial response rate of 45.2%. The effect of different types of hormone therapy was not the same, and the reaction rate of aromatase inhibitors was 100%, while that of tamoxifen was 0%. This patient developed tumor progression after letrozole treatment for 6 months, and there is no previous literature on the treatment of letrozole-resistant GCTs.

Estrogen stimulates the proliferation of granulosa cells by increasing the responsiveness of cells to FSH. Hormonal manipulation of GCTs arises from the surmise that suppression of endogenous estrogen will provide an anti-proliferative milieu that could be effective in treating GCTs. Several mechanisms have been suggested to explain how hormone manipulation may inhibit tumor growth in GCTs. These can be categorized as indirect action on tumors via suppression of gonadotropins or endogenous steroids and direct effects on the tumor via a local mechanism mediated by specific receptors in the GCTs. Various drugs such as medroxyprogesterone acetate, megestrol acetate, tamoxifen, aromatase inhibitors, and GnRH agonists have been tried, but with varied rates of response.^[8]^ Progestins act as chemopreventive agents by inducing apoptosis pathways involving transforming growth factor α (TGF-α) in the ovarian epithelium, a plausible local mechanism for inhibiting tumor growth. Malikh et al^[9]^ have documented prolonged remission (14–42 months) in patients with extensive disease treated with high doses of medroxyprogesterone acetate (100–300 mg thrice daily). Hardy et al^[10]^ used alternating biweekly cycles of megestrol acetate (40 mg twice daily) with tamoxifen (10 mg twice daily) in a patient with recurrent ER-negative PR-positive GCTs documented a CR at 22 months and a DFI of 5 years. Continuous progesterone exposure leads to the depletion and downregulation of PRs in target tissues, while tamoxifen increases PR concentration. Thus, sequential therapy may prolong the antiproliferative effects of progestin by allowing regeneration and stimulation of PRs. Steroidal aromatase inhibitors (anastrozole and letrozole) act by inhibiting the conversion of androstenediol to estriol and testosterone to estradiol. They reduce the aromatization of androgens by up to 90% and have few side effects. Freeman et al ^[11]^ reported the use of anastrozole (1 mg/day) and letrozole (2.5 mg/day) in recurrent GCT and documented remissions ranging from 12 to 54 months. There was a reduction in the size of the disease; few cases had a complete response, and a fall in inhibin levels was observed. Moreover, there was an improvement in performance status. GCTs express receptors for follicle-stimulating hormone (FSH), which supports the growth of GCTs. Thus, hormonal therapies that can decrease gonadotropins may block the stimulatory effects on granulosa cells. Kim et al^[12]^ described PR with monthly GnRH agonists (leuprolide acetate 3.75 mg IM) lasting 3 to 11 months. A few other studies have shown partial response to GnRHa.^[13]^ However, other studies have shown no response to GnRHa.^[14]^ Experimental treatment with Diphereline for this patient was implemented, which has achieved a good therapeutic effect. Prospective multicenter trials are needed to address the role of hormone therapy in the management of these rare neoplasms.

## 4 Conclusion

The surgical treatment of GCTs should aim for optimal cytoreduction, and hormone therapy may be an alternative to recurrent GCTs. It is difficult and time-consuming to study new drugs and combinations in prospective clinical trials because of its relative rarity and prolonged disease course. Large international clinical trials are needed to validate new treatment strategies for patients with GCTs.

## Acknowledgments

The authors thank the Department of Pathology at the Fifth Affiliated Hospital of Sun Yat-sen University for assistance with pathological diagnosis.

## Author contributions

All authors analyzed and interpreted the patient data according to histological examination and literature review. YH was a major contributor in writing the manuscript, and ZY was responsible for the final approval of the version to be published. ZY, ZSS, LY, and YH performed the surgery, and ZY served as the endocrinology doctor who was responsible for the patient's treatment throughout this process. All authors read and approved the final manuscript.

**Investigation:** Shushan Zhang, Yao Liu.

**Methodology:** Shushan Zhang, Yao Liu.

**Supervision:** Yuan Zhuang.

**Writing – original draft:** Hua Yang.

**Writing – review & editing:** Hua Yang, Yuan Zhuang.
